# A Novel *PRPF31* Mutation in a Large Chinese Family with Autosomal Dominant Retinitis Pigmentosa and Macular Degeneration

**DOI:** 10.1371/journal.pone.0078274

**Published:** 2013-11-11

**Authors:** Fang Lu, Lulin Huang, Chuntao Lei, Guiquan Sha, Hong Zheng, Xiaoqi Liu, Jiyun Yang, Yi Shi, Ying Lin, Bo Gong, Xianjun Zhu, Shi Ma, Lifeng Qiao, He Lin, Jing Cheng, Zhenglin Yang

**Affiliations:** 1 Center for Human Molecular Biology & Genetics, The Institute of Laboratory Medicine, Sichuan Academy of Medical Sciences & Sichuan Provincial People's Hospital, Chengdu, Sichuan, China; 2 Sichuan Translational Research Hospital, Chinese Academy of Sciences, Chengdu, Sichuan, China; 3 Department of Ophthalmology, Sichuan Academy of Medical Sciences Affiliated Hospital and Sichuan Provincial People's Hospital, Sichuan, China; 4 Dechang County Hospital of Traditional Chinese Medicine, Liangshan Autonomous Prefecture, Sichuan, China; Sanjay Gandhi Medical Institute, India

## Abstract

**Purpose:**

This study was intended to identify the disease causing genes in a large Chinese family with autosomal dominant retinitis pigmentosa and macular degeneration.

**Methods:**

A genome scan analysis was conducted in this family for disease gene preliminary mapping. Snapshot analysis of selected SNPs for two-point LOD score analysis for candidate gene filter. Candidate gene *PRPF31* whole exons' sequencing was executed to identify mutations.

**Results:**

A novel nonsense mutation caused by an insertion was found in *PRPF31* gene. All the 19 RP patients in 1085 family are carrying this heterozygous nonsense mutation. The nonsense mutation is in *PRPF31* gene exon9 at chr19:54629961-54629961, inserting nucleotide “A” that generates the coding protein frame shift from p.307 and early termination at p.322 in the snoRNA binding domain (NOP domain).

**Conclusion:**

This report is the first to associate *PRPF31* gene's nonsense mutation and adRP and JMD. Our findings revealed that *PRPF31* can lead to different clinical phenotypes in the same family, resulting either in adRP or syndrome of adRP and JMD. We believe our identification of the novel “A” insertion mutation in exon9 at chr19:54629961-54629961 in *PRPF31* can provide further genetic evidence for clinical test for adRP and JMD.

## Introduction

Retinitis pigmentosa (RP) and macular degeneration (MD) are a clinically and genetically heterogeneous group of retinal dystrophies characterized by the progressive degeneration of photoreceptors, eventually resulting in severe visual impairment or blindness [1]. RP and MD are typically characterized as types of rod-cone dystrophy that are caused by the cell death of rod and cone photoreceptors. RP is characterized by a loss of peripheral vision, whereas MD is characterized by a loss of central vision. RP can be divided into autosomal dominant, autosomal recessive, and X -linked hereditary types [2]. The global incidence of RP is about 1/3,500, and more than 100 million people are affected worldwide [3]. MD can have a dominant or recessive inheritance pattern. MD or age-related macular degeneration (AMD) is a leading cause of vision loss in those over the age of 55 years. Juvenile macular degeneration (JMD) is a rare disease that causes central vision loss, often beginning in childhood or young adulthood. Forms of JMD include Best disease, Stargardt's disease, and juvenile retinoschisis [4]. Until now, there have been no effective measures for RP and MD prevention and treatment.

Autosomal dominant RP (adRP) is a common inheritance model of RP. Thus far, 19 loci, including 18 genes, have been identified as adRP-causing genes (RetNetweb site, https://sph.uth.edu/retnet/sum-dis.htm); they are *BEST1* (11q12.3), *CA4* (17q23.2), *CRX* (19q13.32), *FSCN2* (17q25.3), *GUCA1B* (6p21.1), *IMPDH1* (7q32.1), *KLHL7* (7p15.3), *NR2E3* (15q23), *NRL* (14q11.2), *PRPF3* (1q21.2), *PRPF6* (20q13.33), *PRPF8* (17p13.3), *PRPF31* (19q13.42), *PRPH2* (6p21.1), *RDH12* (14q24.1), *RHO* (3q22.1), *ROM1* (11q12.3), *RP1* (8q12.1), *RP9* (7p14.3), *RPE65* (1p31.2), *SEMA4A* (1q22), *SNRNP200* (2q11.2), *TOPORS* (9p21.1) and RP63 (6q23, genesremain to be identified). Among these genes, *RHO* and *PRPF31* are the genes in which mutations are most commonly found in the Chinese population. In the RetNet database, there are also 26 loci, including 23 genes have been identified as being involved in autosomal recessive RP: (arRP) (*ABCA4*, *BEST1*, *C2ORF71*, *C8ORF37*, *CERKL*, *CLRN1*, *CNGA1*, *CNGB1*, *CRB1*, *DHDDS*, *EYS*, *FAM161A*, *IDH3B*, *IMPG2*, *LRAT*, *MAK*, *MERTK*, *NR2E3*, *NRL*, *PDE6A*, *PDE6B*, *PDE6G*, *PRCD*, *PROM1*, *RBP3*, *RGR*, *RHO*, *RLBP1*, *RP1*, *RPE65*, *SAG*, *SPATA7*, *TTC8*, *TULP1*, *USH2A*, and *ZNF513*). Five loci, including three genes (*OFD1*, *RP2*, and *RPGR*), have been identified as being involved in X-linked RP.

There are eleven genes that have been identified as being involved in autosomal dominant MD (adMD) (RetNet website), including *BEST1*, *C1QTNF5*, *EFEMP1*, *ELOVL4*, *FSCN2*, *GUCA1B*, *HMCN1*, *PROM1*, *PRPH2*, *RP1L1,* and *TIMP3*. Two genes, *ABCA4* and *CFH,* have been identified as being involved in autosomal recessive MD (arMD); *RPGR* have been identified as being involved in X-linked MD. In addition, genes *ABCA4*, *ARMS2*, *C2*, *C3*, *CFB*, *CFH*, *ERCC6*, *FBLN5*, *HMCN1*, *HTRA1*, *RAX2*, *TLR3*, and *TLR4* are associated with AMD.

In this study, we reported on a disease-causing gene in a large Chinese family 1085. In this family, 19 patients showed typical clinical symptoms of RP. Among these, five subjects showed RP syndrome and mild MD.

## Materials and Methods

### Ethics Statement

This project was approved by the ethics committee of the hospital of Sichuan Academy of Medical Sciences and Sichuan Provincial People's Hospital, Chengdu, Sichuan, China. Informed consent was obtained from all patients and family members involved in this study. A written informed consent was obtained from each participant.

### Patient Recruitment

The 1085 family members with adRP were collected from Sichuan province. Family members were clinically diagnosed at the Sichuan Provincial People's Hospital. Peripheral blood samples of index cases and their family members were collected in EDTA tubes. Genomic DNA was extracted from peripheral blood by using the standard genomic DNA extraction method.

### Genetic Analysis

#### Linkage analysis

Genome-wide screening was conducted using Linkage analysis chip01 (illumina) according to the protocol. All 1085 family samples were screened. Data set was analyzed using LINKAGE package.

#### Snapshot analysis

Seven SNPs around gene *PRPF31* were selected for Snapshot analysis for fine chromosomal localization. The procedure of Snapshot analysis was carried out according to ABI PRISMSNaPshot™MultiplexKit protocol, and the processed samples were analyzed via an ABI 3130XL genetic analyzer.

#### Sanger sequencing analysis

To find mutations in the disease candidate gene, we used sanger sequencing analysis. The procedure was carried out according to the ABI BigDye sequencing protocol, and the processed samples were sequenced via an ABI3130XL genetic analyzer.

### Clinical diagnosis

Ophthalmic examinations were executed, including of visual acuity, intraocular pressure, ocular motility, pupillary reaction, slit-lamp examination, dilated fundus examination and visual electrophysiological testing. SD-OCT was examined using SPECTRALIS® platform (Heidelberg engineering, Germany). mfERG was detected using RetIscan (Roland instruments, Germany).

## Results

### Clinical Manifestations of Members of the Pedigree

**Figure 1:**
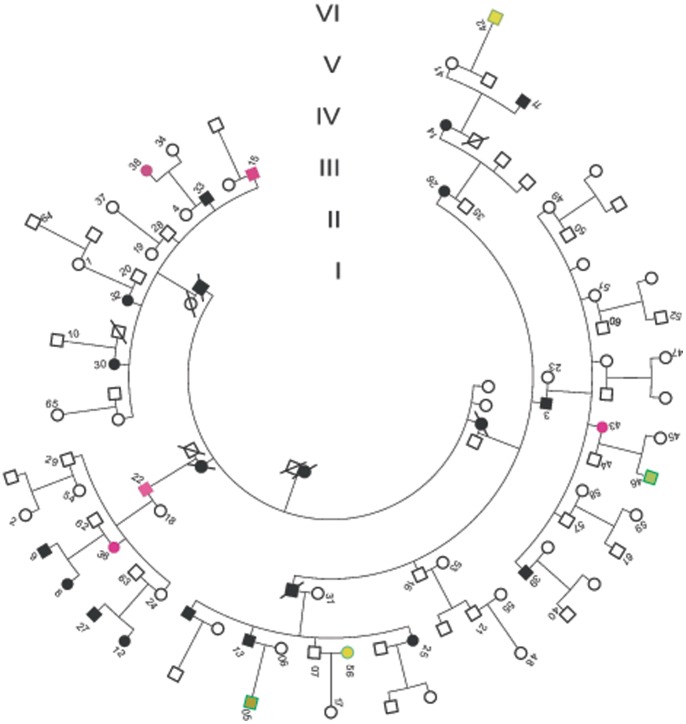
The pedigree of the family 1085, with autosomal dominant retinitis pigmentosa. Normal individuals are shown as clear circles (female) or squares (male), and affected individuals are shown as solid symbols. Patients with fovea centralisareflexia are highlighted in red. This family contains six generations in total (shown in Roman numerals). Individuals with the *PFPR31* gene mutation in the form of incomplete penetranceare are shown in green (samples 5, 42, 46, and 56).

**Figure 2:**
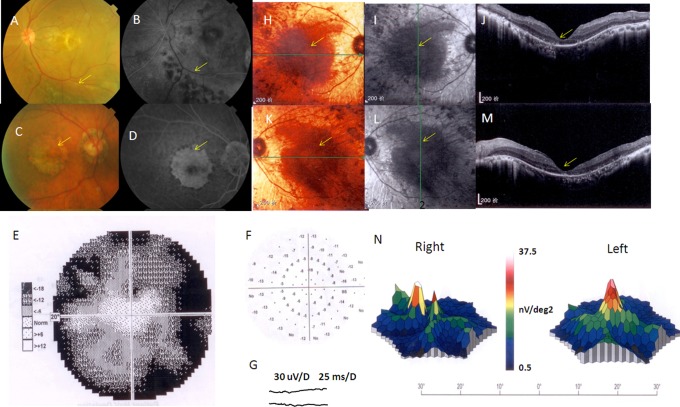
Images of subjects 13, 38 and 35 from the family 1085. A and B are color fundus photographs and black-and-white fluorescein angiograms (FA) of subject 13. Clinical changes were essentially identical for both eyes. Only the left eyes are shown here. The arrows point to abundant pigmentation. C and D are color fundus photographs and FA of subject 38, who was affected by RP syndrome and JDM. Clinical changes were identical for both eyes. Only the right eyes are shown here. In this case, the pigmentation revealed macularatrophy. The arrows point to pigmentation around the macular and macular atrophy. E and F are the visual fields of subject 38. G is the ERG response of subject 38; no A or B waves could be detected. H and I, right eye macular fundus figures of subject 15. J, OCT of subject 15′s right eye. The arrows directed for the macular pathology. K and L, the left eye macular fundus colored and black-and-white figures of subject 15. M, OCT of subject 15′s left eye. N, right and left eyes' mfERG pictures of subject 15.

In this six-generation pedigree 1085, 65 members consented to participate in this study (Fig. 1). Nineteen individuals of the 1085 family were considered to be affected by RP, and 46 individuals were considered to be unaffected. A fundus examination of the patients showed typical RP features, including peripheral vision loss, night blindness, optic disk atrophy, retinal vascular stenosis, pigmentation, and the severe reduction or extinguishing of ERG. For example, typical pigmentation can be seen on the retina of subject 13, who experienced RP onset during his childhood (Fig. 2 A, B). An exception was subject 22, who experienced RP onset at the age of 48. The other patients experienced RP onset during their childhoods. Furthermore, patient 38 exhibited features of macular degeneration in her childhood or young adulthood (Fig. 2 C–G). Similar fovea centralisareflexia phenotype can be found in patients 15, 22, 36 and 43. For example, patient 15, both his right eye and left eye in macaia showed attenuation by SD-OCT examination (Fig. 2 H–M) and he has very low light stimulus reaction by mfERG examination (Fig. 2 N), especially in his right eye. Patients with fovea centralisareflexia are highlighted in red in Fig. 1. In addition to JMD, subjects 3, 14, 15, 25, 26, and 32 showed bilateral cataractvia by slit lamp examination. The detailed information on the affected patients is shown in Table 1.

**Table 1:** Features of the 1085 pegigree patients.

Subject	Gender	Age	Height (cm)	Weight (kg)	On-set of RP	Present VA	clinical symptoms
1085-03	Male	82	173	65	child	<<0.1	RP, bilateral cataract
1085-08	female	13	139	26	child	<<0.1	RP
1085-09	Male	15	144	32	child	<<0.1	RP
1085-11	Male	19	170	55	child	0.2/0.3	RP
1085-12	female	19	163	50	child	<<0.1	RP
1085-13	Male	41	174	81	child	<<0.1	RP
1058-14	female	43	160	57	child	0.3/0.3	RP, bilateral cataract
1085-15	Male	51	174	50	child	0.4/0.6	RP, fovea centralis areflexia, bilateral cataract
1085-22	Male	65	178	58	48	<<0.1	RP, fovea centralis areflexia
1085-25	female	43	152	67	child	<<0.1	RP, bilateral cataract
1085-26	female	68	159	50	child	<<0.1	RP, bilateral cataract
1085-27	Male	17	165	45	child	<<0.1	RP
1085-30	female	53	158	65	child	0.2/0.3	RP
1085-32	female	55	160	43	child	0.1/0.1	RP, bilateral cataract
1085-33	Male	46	177	74	child	0.5/0.5	RP
1085-36	Female	39	165	51	child	<<0.1	RP, fovea centralis areflexia
1085-38	Female	21	170	55	child	<<0.1	RP, fovea centralis areflexia, JMD
1085-39	Male	35	180	90	8 to 9	N	RP
1085-43	Female	37	160	60	child	N	RP, fovea centralis areflexia

### Mutation Screening and SNP Genotyping Results

First, via genome-wide scan and Linkage analysis, rs9788, rs8109631, rs465169, rs36633, and rs8111838 in 19q13.42 were shown to be associated with RP in the 1085 family. In this locus, only the *PRPF31*gene is reported to be an adRPdisease-causing gene. Then, we selected seven SNPs around the *PRPF31* gene, including rs4806711, rs56220912, rs10424816, rs254271, rs8109631, rs465169, and rs36633, for Snapshot analysis, using all 1085 family samples (primers designed for SNaPshot analysis are shown in Table 2). Via Snapshot and Linkage analysis, the maximum two-point LOD score is 2.64 at θ = 0.1 at rs10424816 (Table 3). This result suggested that the *PRPF31* gene might be the disease-causing gene for the 1085 family. We next sequenced the complete exons and the flanking regions of the *PRPF31* gene.

**Table 2:** Primers used for Snapshot analysis.

SNP		Primer	Size
rs4806711	F	ACGTGAGTCCCTTTCCTCCT	506bp
	R	GGGGAAACCCCGTCTCTACT	
Snapshot primer		AGGAGAGGTGAGTGTGATGG	
rs56220912	F	GCCAACCAGCAGAGTCTACC	504bp
	R	CCTCTCCAGCTCTCTGCACT	
Snapshot primer		GCTCACTCTCGGACCCCCTC CCAGAGGCCT	
rs10424816	F	GGGCGTCTTTTCCTCTGG	423bp
	R	GTTCACTGCAACCTCCGTCT	
Snapshot primer		CTAGGTCTGC TGTTGGAAGGTAGCATGAACCTACTGGCTT	
rs254271	F	GGCTGAAGTCAGGGTGTCAT	407bp
	R	ACAGATCCTGGTGTGGAAGG	
Snapshot primer		TGCTTCTGTCTTCATATCTC	
rs8109631	F	CCCAGATTTGGAGTCAGCAT	428bp
	R	AGGGCTTCTCCCCAGTATGA	
Snapshot primer		CAATCAGATGATCATCAATTATGTCAAAAG	
rs465169	F	ACCCAACCTCACCCTACCTC	500bp
	R	GCTGTGTTCTTGAGCCTTCC	
Snapshot primer		GCTCCCTCCGCTCCGGTCTTCTACCCCAGGGCTGGTCTTT	
rs36633	F	AAGAGACCAGCCCCAGTTCT	523bp
	R	TTGGTGGTTTGAGTCCCTTC	
Snapshot primer		CAGTCATGCTGCACACAGCTGATGACTGGGATGGAGGCATTAGCCCTGGA	

**Table 3:** Two-point LOD scores around disease causing gene *PRPF31.*

SNP	Location (chr19)	θ = 0	θ = 0.1
rs4806711	54619191	−2.24939	0.537837
rs56220912	54626055	1.750628	1.44181
rs10424816	54630208	1.69589	2.641756
rs254271	54630757	−0.78082	1.591041
rs8109631	54080144	1.190325	2.612824
rs465169	54526970	0.598506	2.773549
rs36633	54646288	1.531824	1.285244

**Figure 3:**
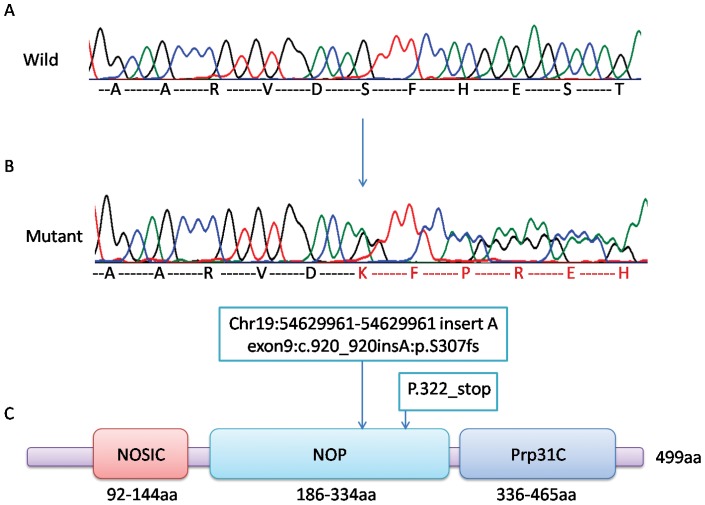
Detected mutation in the *PRPF31* gene. A is the wild type sequence peak chart of the *PRPF31* gene. B is the mutant type sequence peak chart of the *PRPF31* gene: the heterozygous mutation that results in a single “A” nucleotide's insertion at chr19:54629961-54629961 (exon9, c.920_920insA). This insertion leads to the coding protein's frame shift at p.307 and early termination at p.322. C is the predicted *PRPF31* protein's domains, showing that the mutation is in the functional domain NOP, whichis essential for U4/U6-U5 tri-snRNP formation.

We sequenced the exons of *PRPF31* gene, using all members of 1085 family. Primers designed to amplify all 14 exons and flanking regions of *PRPF31* from the genomic DNA are shown in Table 4. We found that a novel heterozygous insertion in exon9 at chr19:54629961-54629961 (UCSC:feb.2009 (GRCH37/HG190)) that inserted an “A” nucleotide was co-segregated between patients and normal members (Figure 3AB). Mutations can be detected in all the affected samples. However, this mutation can also be detected in normal samples 5, 42, 46, and 56, showing partial penetrance (Figure 1 in green). This nonsense mutation leads to a protein frame shift from p.307andearly termination at p.322 in the snoRNA binding domain (NOP domain) (Figure 3C).

**Table 4:** Primers used for *PRPF31* gene whole exons sequcencing.

Exon		Primer	Size
Exon1	F	AGTTTCCTGTTTCCGGCTTC	437bp
	R	TAAAGACCCGCCTTTTTCCT	
Exon2	F	TTTGTCGGGGCAAGTTTTTA	300bp
	R	AAGCCTGTATCACCCCCTTC	
Exon3	F	TAGCAGGGGGCTCTAGACAG	203bp
	R	GCAGGAGAGACAGGAGATGG	
Exon4	F	CGAGAGGGGGTAGGGATTTA	214bp
	R	GAAAGGCCAGTGGGGAAG	
Exon5	F	AAAGGAAGAAGGGGACATGG	214bp
	R	AGAAGCACCCCACCTTCTCT	
Exon6	F	AGGAGGTGCTGAGCAAGAGA	250bp
	R	CGTGTGTAGCTCCAGCCTAA	
Exon7	F	CAGGTGTACACACGCACACA	432bp
	R	GCTGACCTCTGTGATGTCCA	
Exon8	F	TACTCACCCCCACCTCTCTG	299bp
	R	GTGGCTGCTCAGGCTGTC	
Exon9	F	CGGTTGCTTTGCTGTTACCT	209bp
	R	CAGGCCCAGAGGAAAAGAC	
Exon10	F	TTTAACTAAGGCACGTGGATACTC	267bp
	R	CATGACCCCCATGCCTAC	
Exon11	F	GGTAGGCATGGGGGTCAT	250bp
	R	GCCACAGGACGAGAGGAG	
Exon12	F	TAGATCGAGGAGGACGCCTA	207bp
	R	ACAGGGAGGCTGCGATCT	
Exon13	F	ACCGAGGGACACAAGGTG	244bp
	R	CTCATCCTGGCCTTCTTCAC	
Exon14	F	GGCTCTGATGGGTCACAGTT	514bp
	R	CCGGCTGTTTGAAAAATGAT	

## Discussion

In our pedigree, we found one novel *PRPF31*mutation in a large adRP family. In this family, subject 38 showed adRP and JMD. Subjects 15, 22, 36, and 43 showed both adRP and fovea centralisareflexia. According to our findings, we propose that the *PRPF31* gene is the gene that causes adRP and MD. Although six subjects showed bilateral cataracts, it is difficult to diagnose the genetic factors of cataracts for RP patients over 40 years of age. RP and MD are the most common degenerative diseases of the retina. To date, about eight genes have been identified as disease-causing genes for patients with RP and/or MD. For example, mutations in ABCA4, the photoreceptor ABC transporter, are associated with Stargardt macular degeneration [5] and arRP [6]–[7]. Mutations in *BEST1* can cause multifocal Best vitelliform MD (Best disease) [8] and adRP [9]. Mutations in *FSCN2* [10]–[11] and *PRPH2* (*peripherin/RDS*) [12]–[13] can cause adMDand adRP. Mutations in *PROM1* [14]–[15], *RP1* [16], *RPE65* [17]–[19], and *RPGR* [20]–[21] can also cause MD and/or RP.

The *PRPF31*gene codes for the splicing factor hPRP31. Mutations in *PRPF31* have been repeatedly found to be associated with autosomal dominant retinitis pigmentosa (adRP). In 1994, an adRP locus on 19q13.4 (RP11) was first localized via Linkage analysis in a large British family [22]. Then, within this region, mutations in the *PRPF31* gene were identified in other families and sporadic cases [23]. Mutations in *PRPF31* are inherited in an autosomal dominant manner, accounting for about 5% of cases of adRP [1]. Additionally, genomic rearrangements of the *PRPF31* gene account for about 2.5% of adRP cases [24]–[25]. Various mutations have been identified in the *PRPF31* gene that are associated with adRP, including 769–770insA [26], the in-frame deletion of four amino acids 111–114 [27], splice site mutation (IVS8+1G>C) [28], A194E, A216P [29], and c. 1142 del G [30].

*PRPF31* is one of the three pre-mRNA splicing factors that encode components of thespliceosomeU4/U6*U5 tri-snRNP [31], which has been identified as causing adRP (the other two genes are PRPF3 and PRPF8) [26]. This complex can excise introns from RNA transcripts. The disease mechanism for RP11 is caused by mutations in the splicing factor gene *PRPF31* because its' splicing function is incomplete [32]. These spliceosome proteins are highly conserved in eukaryotes ranging from mammals to yeast.

The underlying mechanism via which *PRPF31* causes adRP and MD is still unknown. The inheritance pattern of *PRPF31* mutation is atypical of dominant inheritance [33], which suggests partial penetrance: a dominant mutation appears to “skip” generations. A significant difference in wild-type and mutant *PRPF31* mRNA levels was observed between symptomatic and asymptomatic individuals; this can partially explain the incomplete penetrance phenotype of adRP caused by *PRPF31* mutation [34]. However, there are probably more subtle molecular mechanisms underlying this disease [35]. In zebrafish, it was suggested that distinct mutations in *PRPF31* can lead to photoreceptor degeneration via different mechanisms, such as haplo-insufficiency or dominant-negative effects [36].

The *PRPF31*gene codes for 499 amino acids (55 kD). The *PRPF31* protein contains three domains: NOSIC (NOSIC NUC001 domain, from 92aa to 144aa), NOP (snoRNA binding domain, from 186aa to 334aa), and Prp31C (terminal domain, from 336aa to 465aa). Previously, yeast two-hybrid analysis result had shown that the NOP domain is a genuine RNP-binding module, exhibiting RNA- and protein-binding surfaces [37]. In this study, we found a frame shift insertion mutation in the NOP domain area. This mutation causes the protein frame shift at p.307 and early termination at p.322 after coding for 15 missense amino acids. This nonsense protein suggested aberrant hPrp31-hPrp6 interaction that blocks U4/U6-U5 tri-snRNP formation, which may be the reason that the 1085 family was affected by adRP and MD.

In summary, we have, for the first time, identified a heterozygous insertion in exon9 at chr19:54629961-54629961 (UCSC:feb.2009 (GRCH37/HG190)), inserting an “A” nucleotide mutation in the *PRPF31* gene, causing typical adRP and JMD. Our study provides evidence that a mutation in the *PRPF31* gene is related, at least partially, to the pathogeneses of both adRP and JMD.
